# Laparoscopic ureteroneocystostomy for iatrogenic ureterovaginal fistula after modified radical hysterectomy: A case report

**DOI:** 10.1016/j.eucr.2022.102144

**Published:** 2022-06-27

**Authors:** Yuichi Machida, Hiroki Yoshiuchi, Yuko Kitano, Masato Kamizuru, Junji Uchida

**Affiliations:** a Department of Urology, Yao Municipal Hospital, 1-3-1, Ryuge-cho, Yao-city, Osaka, 581-0069, Japan; b Department of Urology, Osaka Metropolitan University Graduate School of Medicine, 1-4-3 Asahi-machi, Abeno-ku, Osaka, 545-8585, Japan

**Keywords:** Ureterovaginal fistula, Laparoscopic ureteroneocystostomy

## Abstract

An unique laparoscopic ureteroneocystostomy technique was performed to treat an iatrogenic ureterovaginal fistula that was formed in a 69-year-old woman following open modified radical hysterectomy for endometrial cancer. Severe adhesions between the distal ureter and the surrounding tissues, including the iliac artery, were observed. Owing to difficulties in identifying the distal ureter, the proximal ureter was identified and dissected downward to free the ureter, thereby allowing anastomosis. This report shows that laparoscopic ureteroneocystostomy for ureterovaginal fistula repair may prove useful owing to its minimally invasive and broad approach.

## 1 Introduction

Iatrogenic ureteral injuries are a common occurrence during various pelvic and gynecologic procedures. The prevalence of ureteral injuries after gynecologic surgeries was reported to range from 0.3% to 2%.^1^ Although early and consecutive management using a ureteral stent might be the most appropriate initial approach because of the minimally invasive nature of the technique, a successful endoscopic indwelling of the stent might prove challenging because of the complexity of the ureteral injury. Early surgery is recommended because it can reduce the possibility of renal function damage caused by ureteral stenosis, shorten the treatment time, reduce the cost of hospitalization, and eliminate the physical and mental anguish of patients with ureterovaginal fistulas. Minimally invasive surgical interventions, such as laparoscopic ureteroneocystostomy, may prove feasible and effective for treating ureterovaginal fistulas. Here, we report a case of laparoscopic reimplantation of the ureter in a patient who developed a ureterovaginal fistula following hysterectomy for endometrial cancer.

## 2 Case presentation

A 69-year-old woman underwent subtotal hysterectomy, bilateral salpingo-oophorectomy, and bilateral lymphadenectomy for endometrial cancer. The patient was referred to the department 3 months after surgery for a suspected genitourinary fistula that developed due to persistent vaginal effusion after the postoperative period. Cystoscopy confirmed the absence of a vesicovaginal fistula; however, contrast accumulation in the space between the ureter and the vagina was observed via contrast-enhanced computed tomography, and a ureterovaginal fistula was suspected. Retrograde urography and ureteroscopy were attempted to confirm the diagnosis, but a guide wire could not be inserted through the ureteral orifice because the ureter was blind-ended. Ultrasound-guided percutaneous antegrade pyelography showed that the contrast medium directly leaked from the distal ureter into the vagina, thereby leading to a diagnosis of a ureterovaginal fistula. Indwelling of the nephrostomy tube was not required due to the absence of any obvious signs of infection; a laparoscopic ureteroneocystostomy was planned 3 weeks after the definitive diagnosis.

### 2.1 Surgical procedure

**Fig. 1:**
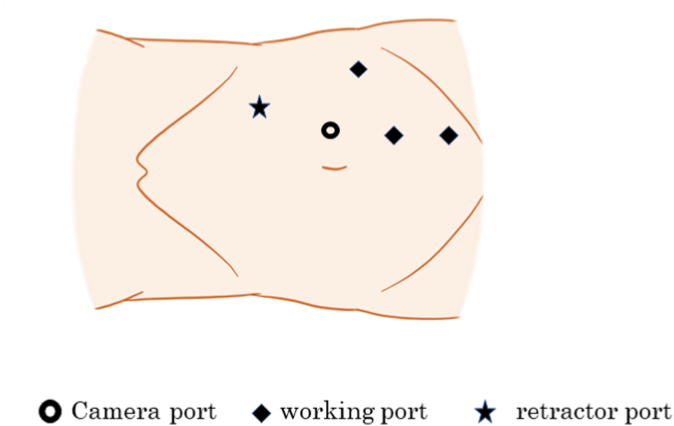
Port placements for left-sided ureteroneocystostomy.

**Fig. 2:**
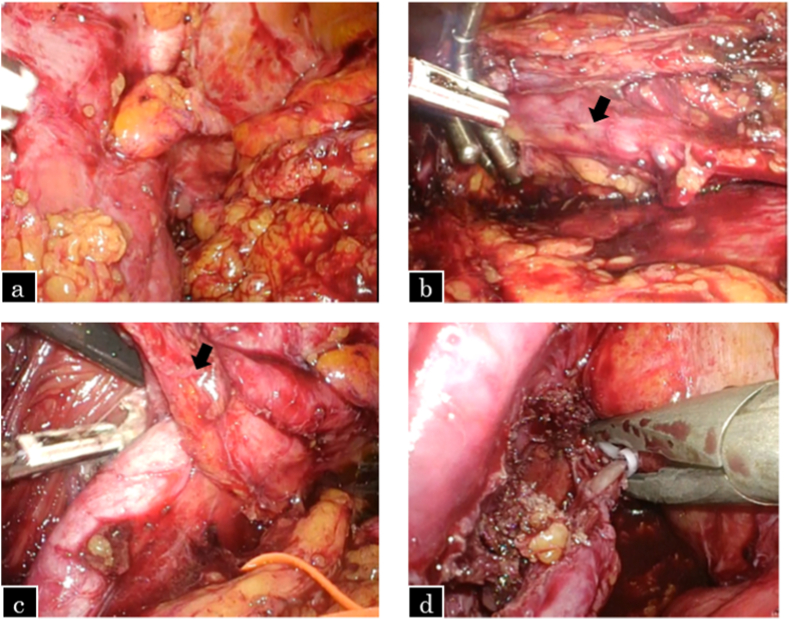
Intraoperative findings. a. The scarred area around the injured distal ureter.b. The proximal ureter (arrow) lifted by the retractor.c. Dissection between the strongly adherent iliac artery and the ureter (arrow).d. Clipping the end of the distal ureter.

**Fig. 3:**
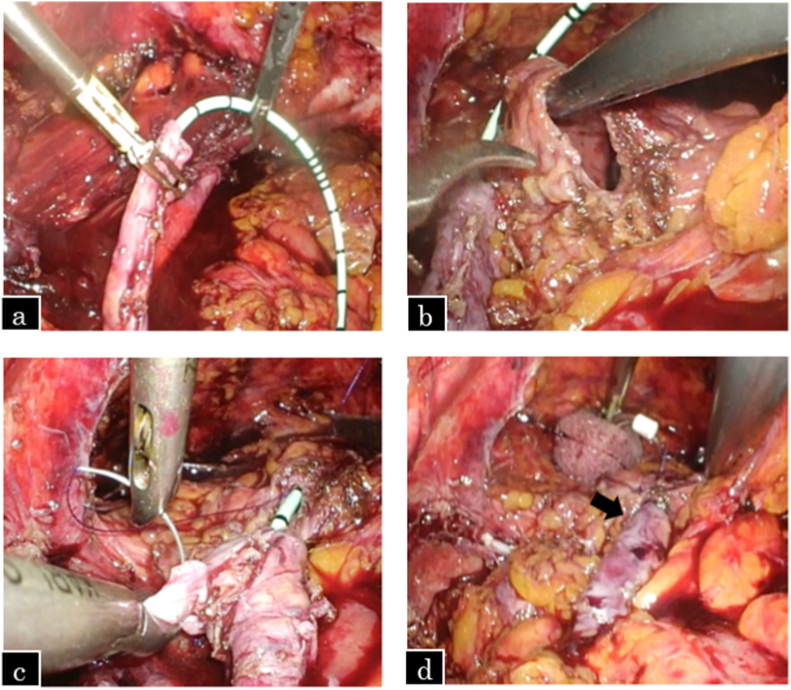
Intraoperative findings.a. Ureteral stent being placed.b. Bladder opened.c. Ureter anastomosed to the bladder.d. The end of the anastomosis and submucosal tunnel (arrow).

The patient was placed in the right lateral position with a 45° tilt, and five ports were used. A 12 mm balloon trocar for the camera port was placed at the outer edge of the rectus abdominis muscle at the level of the umbilicus, as shown in Fig. 1. The previous surgery had resulted in extensive inflammatory adhesions extending from the space of the Retzius to the common iliac lymph node area. Owing to difficulties in identifying the distal ureter, the lower pole of the kidney was elevated with a retractor to identify the proximal ureter, which was then dissected downward to the distal ureter. The ureter and common iliac artery were strongly adehered due to lymph node dissection during the previous surgery; therefore, the patient had difficulty in dissecting the ureter in the crossing portion. The ureter was dissected until just above the scarred area and transected as distally as possible. The normal healthy proximal part was spatulated at the six o’ clock position. The bladder was filled with sterile saline and adequately mobilized from Retzius. A ureteral stent was passed proximally into the ureter and distally into the bladder, and the ureter was anastomosed to the anterolateral wall of the bladder using 4-0 polydioxanone (PDS) sutures using the Lich-Gregoir extravesical reimplantation technique. The detrusor was buttressed on top of the ureter, forming a submucosal tunnel using 3-0 Vicryl. The psoas hitch procedure was not performed owing to the absence of any tension on the anastomosis. The intraoperative findings of the patient are shown in Fig. 2, Fig. 3.

No intraoperative or postoperative problems were encountered. The urethral Foley catheter and stent were removed 2 and 6 weeks, respectively, after the surgery. The subsequent course was uneventful, and the patient was quite satisfied.

## 3 Discussion

In this report, we demonstrated the use of a unique laparoscopic ureteroneocystostomy method for an iatrogenic ureterovaginal fistula that developed following an open radical hysterectomy procedure. No obvious complications were encountered during and after the procedure. Minimally invasive surgical interventions, such as laparoscopic ureteroneocystostomy might be feasible and effective for treating a ureterovaginal fistula. Endoscopic indwelling of the ureteral stent may lead to the recurrence of ureteral stricture after the removal of the stent. Early surgical intervention using a minimally invasive technique might prove beneficial for treating the ureterovaginal fistula caused during obstetric and gynecological procedures, such as hysterectomy for gynecological malignant tumors.

In the current case study, a delayed ureterovaginal fistula was detected after open hysterectomy and lymph node dissection. Despite difficulties in dissecting the tissues around the ureter intraoperatively due to the expected severe adhesion in the retroperitoneal cavity, the minimally invasive repair was accomplished laparoscopically. By placing the patient in a lateral position and devising port placement, the ureter was identified at the lower pole of the kidney to avoid misidentification and dissected without causing any damage. To date, Several case reports on pure laparoscopic or robot-assisted ureteroneocystostomy for ureterovaginal fistula have been reported, but most of them were performed in the supine position.^2^, ^3^, ^4^, ^5^ Supine position has the advantage of facilitating anastomotic manipulation, but the approach to the proximal ureter is difficult. The ureter can be freed over a long distance by proceeding with the dissection from the proximal ureter, thereby allowing for the anastomosis without any tension at the anastomosis site. If the length of the available ureter is shorter than expected because of the extent of the injury, anastomosis may be possible by dissecting the head side of the kidney and using a laparoscopic maneuver to move the kidney caudally or by using the psoas hitch procedure.

Thus, laparoscopic surgery for ureterovaginal fistula repair may be considered useful owing to its minimally invasive and broad approach. Performing the laparoscopic maneuver in the lateral position might allow access to the proximal ureter, which is useful for the identification and dissection of the ureter.

## 4 Conclusion

Ureterovaginal fistula is one of the most serious urinary tract complications that can affect the quality of life of the patient. Laparoscopic ureteroneocystostomy may be considered as a safe and feasible option, particularly in cases where conservative treatment has failed.

## Funding sources

This research did not receive any specific grant from funding agencies in the public, commercial, or not-for-profit sectors.

## Consent

The patient gave informed consent to publish images and details in the study.

## Declaration of competing interest

None declared.

## References

[1] Leonard F., Fotso A., Borghese B. (2007). Ureteral complications from laparoscopic hysterectomy indicated for benign uterine pathologies: a 13-year experience in a continuous series of 1300 patients. Hum Reprod.

[2] Modi P., Goel R., Dodiya S. (2005). Laparoscopic ureteroneocystostomy for distal ureteral injuries. Urology.

[3] Pompeo A., Molina W.R., Sehrt D. (2013). Laparoscopic ureteroneocystostomy for ureteral injuries after hysterectomy. J Soc Laparoendosc Surg.

[4] Sharma S., Rizvi S.J., Bethur S.S. (2014). Laparoscopic repair of urogenital fistulae: a single centre experience. J Minimal Access Surg.

[5] Laungani R., Patil N., Krane L.S. (2008). Robotic-assisted ureterovaginal fistula repair: report of efficacy and feasiblity. J Laparoendosc Adv Surg Tech.

